# Metallurgical and Mechanical Property Investigation of ETP Cu Similar Hybrid Friction Welds for Nuclear Fusion Applications

**DOI:** 10.3390/ma19194066

**Published:** 2026-09-23

**Authors:** Michail Dellepiane, Laurie da Silva, Athanasios Toumpis

**Affiliations:** 1 Department of Mechanical & Aerospace Engineering, University of Strathclyde, Glasgow G1 1XJ, UK; 2 Advanced Forming Research Centre (AFRC), University of Strathclyde, Renfrewshire PA4 9LJ, UK; laurie.da-silva@strath.ac.uk

**Keywords:** hybrid friction welding (HFW), ETP copper, solid-state joining, nuclear fusion applications, metallurgical investigation

## Abstract

The evolution of microstructure and mechanical properties in electrolytic tough-pitch (ETP) copper subjected to hybrid friction welding (HFW) was investigated. The effect of introducing a programmable brake on the flywheel’s rotation while maintaining constant friction and forging pressures was explored. The welded joints were examined through a series of investigations, including optical and scanning electron microscopy with electron backscatter diffraction, tensile and microhardness testing, followed by fractography. The optimised weld achieved the highest ultimate tensile strength (≈274 MPa), a 7.87% increase over the base material, and the greatest elongation (~22%), with ductile fractures and visible necking. The measured hardness distribution depicted higher values for the weld zone compared to the thermo-mechanically affected zone, both expressing a significant decrease from the heat-affected zone and base material due to pre-welding strain hardening existing within the copper alloy. EBSD analysis on the weld zone and thermo-mechanically affected zones revealed the presence of refined grains, with low grain orientation spread (GOS) and kernel average misorientation (KAM) values, which indicated the occurrence of dynamic recrystallisation (DRX) mechanisms during HFW. HFW was able to successfully produce ETP Cu-similar welds with 98.1% joint efficiency.

## 1. Introduction

High purity copper is considered a possible candidate material in experimental plasma confinement devices and in applications where high thermal and electrical conductivities (thermonuclear fusion reactors) are required [1,2,3,4]. Copper has a high thermal conductivity of 391.1 W/m·K [5], which is significantly higher than that of conventional structural materials [1]. Its use in high temperature applications helps reduce temperature gradients and the resulting thermal stresses that promote creep and thermal fatigue in structural components [6,7]. However, this same characteristic can cause rapid heat dissipation during conventional welding and can contribute to the formation of large heat affected zones (HAZs), degrading the mechanical performance of the material.

Currently, electron beam welding (EBW), a fusion welding process, is implemented in joining copper and its alloys across various industrial sectors, including aerospace and nuclear power [8,9]. However, it is a joining process that is faced with significant challenges when it comes to copper, such as the formation of defects, equipment costs, and limited field applications due to vacuum requirements [8,10,11]. The growing demand for high-quality joints, combined with the inherent difficulty of welding materials specifically selected for demanding environments, such as nuclear fusion reactors, has created the opportunity to investigate welding processes that can produce true metallurgical welds between similar and dissimilar metals without melting [12,13] in ambient environments with lower cost equipment [14,15]. Amongst these, rotary friction welding (RFW), with its two energy based variants—continuous drive friction welding (CDFW) and inertia friction welding (IFW)—is reported to provide substantial improvements in joint quality compared to conventional fusion welding processes [15,16,17].

Recent investigations [18,19,20,21,22] have demonstrated notable progress in achieving refined microstructures and strong metallurgical bonds on friction welds of copper and its alloys. Successful friction welded (FW) joints exhibited tensile strengths comparable to those of the parent material. Significant grain refinement was observed within the weld zone (WZ), with grain sizes decreasing from approximately 169 µm in the base material (BM) to as little as 20 µm, primarily a result of dynamic recrystallisation (DRX). In these studies [18,19,20,21,22], achieving 100% joint efficiency proved challenging due to a lack of process parameter control and an inability to predict the heat-sink effect of the copper. Furthermore, the bonding mechanisms that control this grain refinement, including the evolution of grain boundaries and the extent of recrystallised fraction, remained rather speculative [19,20,21]. Characterisation techniques such as optical microscopy confirmed grain size fluctuations with limited application of high-resolution electron backscatter diffraction (EBSD) analysis. As a result, the comprehensive understanding of recrystallisation behaviour and the effect of strain on the copper grains remains limited.

These gaps have highlighted the need for a comprehensive microstructural and mechanical property investigation, supported by EBSD mapping, to clarify the dynamic recrystallisation behaviour and its direct influence on hardness, strength, and ductility in friction-welded high grade pure copper alloys. Controlling the welding parameters, such as rotational speed and friction pressure, can have significant effects on the copper’s microstructure and mechanical properties following FW [21]. In this study, the effect of controlling the energy input through a programmable brake was investigated in HFW of electrolytic tough pitch copper (ETP Cu) joints. This study advances the understanding of solid state joining of similar alloys with high thermal conductivity and investigates the effect of weld energy on the integrity of the generated welds.

## 2. Materials and Methods

ETP copper with a nominal chemical composition of ~99.9% Cu was used in this study. The as received rolled copper was machined to cylindrical rods of 35 mm × 100 mm (diameter × length). Welding was carried out using the 125-tonne MTI friction welding machine hosted at the Advanced Research Forming Centre (AFRC). For this study, the HFW cycle invoked the use of a flywheel from IFW as well as the spindle motor from CDFW. In HFW, the flywheel is accelerated to a constant velocity and then held for a part of the welding cycle. The motor is then allowed to coast, therefore enabling the stored kinetic energy to be converted from the flywheel (inertia cycle) into heat, with the break engaging after the specified rpm has been achieved (Table 1). The employed HFW process parameters of the ETP Cu joints are presented in Table 1.

The manufactured joints of ETP Cu were examined following the international standard BS EN ISO 15620 [23] used for friction welding metallic materials. Metallographic samples were machined and prepared according to the standard metallographic preparation methods provided by ASTM E3-16 [24] and etched using a solution of 10% ferric nitride in distilled water. The etched samples were examined under an Olympus GX51 optical microscope (OM) and a Tungsten Filament scanning electron microscopy (SEM) HITACHI S-3700N to evaluate the microstructural evolution across different weld zones. For characterisation with electron backscattered diffraction (EBSD), samples were also subjected to vibratory polishing for two hours in a pool of colloidal aqueous silica solution. The EBSD maps were collected using an Oxford Instruments EBSD detector interfaced to the HITACHI S-3700N SEM at an accelerating voltage of 20 kV and a step size of 0.5 µm with a minimum hit rate of 85%. For all cases, the post-processing of the EBSD data was carried out using the Aztec Crystal software. Tensile tests were carried out at ambient conditions on cylindrical specimens with a gauge diameter of 12.50 mm at a rate of 1 mm/min in accordance with ASTM E8/E8M [25]. Three tensile specimens were tested at each condition for statistical reliability, extracted from the same radial distance of the 35 mm radius workpieces. Vickers microhardness (HV) measurements were taken along the cross-sections of the welds in the form of line profiles in the centre and outer regions, covering BM, heat affected zone (HAZ), thermo-mechanically affected zone (TMAZ) and WZ. A 0.5 HV load was applied over a 10 s dwell time, and measurements were taken along the centre of the welds.

## 3. Results and Discussion

### 3.1. Mechanical Properties

#### 3.1.1. Tensile Testing

Transverse tensile testing was carried out to establish the effect of the chosen process parameters on the HFW ETP Cu joints. The yield strength (YS) and ultimate tensile strength (UTS) against the % elongation for each weld were measured and compared to the parent material properties. Figure 1 presents the averaged tensile behaviour for each series of testing per weld, with statistical average deviation as error bars for YS, UTS and % elongation in accordance with ASTM E8/E8M-25 [25]. The introduction of an applied brake at increments of 25 rpm had a substantial effect on the strength and ductility of the flow-formed weldments. The YS and UTS of the parent material were measured to be ≈248 MPa and ≈250 MPa, respectively.

For welds C1850 and C1875, during tensile testing, the final fracture was observed between the HAZ and TMAZ. However, in the C1900 welds, the final fracture occurred at the WZ. The weld specimens did not demonstrate a distinct yield point, but the yield strength was approximated with the 0.2% offset method as 102 MPa for C1850, 128 MPa for C1875 and 140 MPa for C1900, averaged as per Figure 1. None of the examined welds demonstrated a yield strength above that of the parent material, with similar results found in previous studies [18,19]. However, in comparison to the parent material, weld specimen C1875 exhibited an increased UTS, measured at ≈274 MPa, i.e., a 7.87% increase. This increase in UTS was not recorded for welds C1850 and C1900, with an 8.66% decrease (UTS for C1850 is 232 MPa) and a 15.35% decrease (UTS for C1900 is 215 MPa) for each weld, respectively. Similarly, weld C1875 was recorded to have the highest ductility at approximately 22% elongation compared to 21% of C1875 and 3% of C1900. At constant friction and forging pressures and durations, with the only variable being the implementation of the break, C1875 provides the optimal results. Thus, it can be proposed that the difference in strength of the welded specimens was associated with the initial rotational speed of the friction welding cycle, which, in turn, influenced the weld energy input to the system. With the maximum available rotational speed for the friction welder being 2000 RPM, it was understood that allowing the system to operate for only 100 rpm led to insufficient heat generation and with a lower plastic flow at the weld interface. Increasing the weld energy with a stop at 1875 RPM led to an increase in the interface temperature, generating adequate heat to soften the material for viscous flow and plastic deformation—weld C1875—as also reported by Lu et al. [26]. However, due to copper’s high heat conductivity, it was critical to control the circulation of thermal energy along the axial direction, avoiding a large “heat sink” leading to an extended HAZ and TMAZ. This was evident for weld C1850 where heat dissipated within the welded specimen, softening the weld resulting in lower yield strength and UTS. The weld joint efficiency for weld C1875 was measured to be 98.1%, by calculating the ratio of the strength of the joint versus the strength of the base material, as expressed in Equation (1):

(1) $$Weld\;Joint\;Efficiency=\;\left(\frac{Joint\;Strength}{Base\;Material\;Strength}\right)\;\times 100\%$$

#### 3.1.2. Hardness Distribution

Hardness measurements were recorded at the central region along the AD of the three welded joints and are represented in Figure 2. The supplier’s certificate of conformity provided hardness values in the range of 40–120 HV; however, the hardness variation in the investigated HFW copper workpieces revealed the material’s diverse processing history. Typically, in copper, hardness values near 40 HV indicate that recrystallisation or annealing has taken place [5,27,28], whereas values between 90–120 HV correspond to strain hardening via cold work [29,30,31]. For the present study, the hardness in the BM region of the EPT copper workpieces, was measured to be in the region of 80–90 HV, indicating that pre-processing of the copper alloy has taken place, as also supported by the EBSD results. This follows similarly published studies in which cold worked copper demonstrated higher hardness than the as-received workpieces, due to an increase in both dislocations and grain boundary densities [31].

A similar hardness profile can be observed for all three welds, with a steep decrease taking place at the transition point from BM to the HAZ just before the TMAZ threshold (Figure 2). Initially, a slight hardness decrease is observed due to the recovery process, as temperature increases. Following this, copper started to plastically deform due to exposure to high temperatures, shear and compressive strains, leading to a decrease in hardness from the initial cold worked BM, indicating the occurrence of recrystallisation [32]. In this range, the copper alloy hardness declines to 48.6 HV for C1850, 46.2 HV for C1875 and 49.5 HV for C1900, and then stabilises between 50–60 HV for the TMAZ and WZ. Furthermore, the EBSD KAM maps provided in Section 3.2.3 reveal that the volume density of high angle grain boundaries, a barrier for dislocation motion, increased with increasing strain and temperature. Thus, grains in the TMAZ and WZ have undergone GDRX, forming fine equiaxed grains—as examined in Section 3.2.1—with the slight final increase in hardness attributed to the Hall–Petch effect [28,33].

#### 3.1.3. Fractography of Welded Specimens

Based on the tensile results, fractographic analysis was conducted to relate the mechanical performance of the welded joints to the corresponding fracture mechanisms and weld quality. The macroscopic fracture surfaces of the highest and lowest elongation results, C1875 at 20% and C1900 at 3%, respectively, are presented in Figure 3a,b.

In Figure 3a, the fracture location is identified immediately above the WZ, with red arrows indicating the estimated pre-weld copper substrates interface. A complete separation is observed followed by a secondary separation above the main fracture area. Comparatively, in Figure 3b the fracture location is identified at the HAZ with evidence of gradual necking, including quasi cup and cone characteristics, an indication of ductile fracture. For the case of the flat profile of the C1900 weld, where low elongation is also observed—with welds fracturing at 3% elongation on average, as shown in Figure 1—a further microscopic investigation by SEM is carried out with the microstructural changes featured in Figure 4.

The SEM micrograph exhibits two separate features; ductile micro-dimples (majority of the surface) and flat regions, indicated with red arrows. These regions are fragments of the machined surface finish of the pre-welded cylindrical workpieces that have not undergone plastic deformation, revealing highly brittle behaviour. Similarly, the surface on the outer diameter of each weld, where heat generation is expected to be considerably higher, increased plastic deformation has occurred, which can be considered an indication of higher ductility. This combination of microstructural characteristics is found throughout the fractured surface of C1900, with an increased frequency towards the centre of the weld, where heat generation would be expected to be the lowest [33]. In contrast, these characteristics were not detected in the C1875 and C1850 welds. This further demonstrated the effectiveness of transitioning from IFW to HFW and the level of accuracy required in deciding when to terminate the welding cycle. The observed fracture behaviour demonstrates that even a 25 rpm variation in rotational speed can significantly influence the local deformation conditions and, consequently the final weld quality. The rate of frictional heat generation increases with increasing weld energy, where this heat is necessary to soften the material for plastic deformation to occur [34]. Banerjee et al. [34] described that the amount of heat dissipated or transferred to the adjacent weld regions is directly proportional to the weld energy (or rotational speed) and is expressed by Equation (2) as follows:

(2) $$Q=\;\frac{\pi^{2}PµNR^{3}}{20}$$

where *Q*, *P*, *R*, *N* and μ represent the heat, pressure, radius of the weld component, rotational speed and coefficient of friction, respectively. In this context, C1875 appears to present the most favourable welding parameter set among the three welds investigated. This is supported by its improved mechanical properties and the absence of retained machined surface features at the weld interface.

### 3.2. Microstructural Characterisation

#### 3.2.1. Optical Microscopy

A detailed microstructural characterisation of the best performing weld, C1875, along its axial direction (AD) was carried out to correlate microstructure development to mechanical performance. The macrograph of weld C1875 highlighting the flash generated during HFW with the fixture and the spindle side is shown in Figure 5a,b. Furthermore, Figure 5c provides a top-down view of the metallographically prepared and etched surface with the developed weld regions WZ, TMAZ, HAZ and BM visibly identifiable.

Defects along the weld area are not apparent—compared to similar studies with flat faced copper workpieces [19,20,21]—with the locations of microstructural variations with respect to the unaffected BM being visually observed (marked white dotted lines). Specifically, the TMAZ exhibits significant grain refinement when compared to the BM, which occurred during the welding process. Optical microscopy is introduced with Figure 6a–c, with the annotated weld zones of C1875 revealing grain evolution and their distinct surface morphology.

Grains in the BM appear predominantly homogeneous and equiaxed without a specific directionality. The dark-shaded particles that are present throughout the weld zones are identified as Cu_2_O inclusions. Davis et al. [5] established that the effect of these copper oxide inclusion on mechanical properties are minor. The copper grains significantly change in shape and volume between HAZ and TMAZ, with the heavily deformed WZ not exhibiting any indication of the initial point of contact between the workpieces. Grains in the TMAZ and WZ areas appear strain free and equiaxed and are often found in cases where recrystallisation has ensued [35], with a small volume of anisotropic growth due to the direction of the heat flow [36] that lead to plate-shaped grains. This is due to the high temperatures and strain/deformation experienced in the TMAZ and WZ, which are accompanied by a large volume of twin boundaries.

Copper (α-phase) crystallises in a face-centred cubic (FCC) structure with an intermediate stacking fault energy (SFE) value of 50–60 mJ m^2^ [37]. A direct correlation between SFE and deformation mechanisms in metallographic texture analysis has been reported elsewhere [38,39]. Materials with high SFE, such as aluminium alloys (~170 mJ/m^2^), are known to deform via dislocation slips [40]. Comparatively, twinning is the dominant deformation mechanism in austenitic steels (~20 mJ/m^2^) [41]. Copper is closer to the steel threshold, at which it will primarily deform through dislocation movement along specific slip systems, with twinning serving as a secondary deformation mechanism [42,43,44], especially under high strain rates. The OM micrographs presented in Figure 6 provided a clear indication of the microstructure evolution and the presence of twin boundaries from the BM to the WZ. Humphreys et al. [45] further argued that twin formation can occur throughout recovery, recrystallisation and grain growth during solid-state processes where plastic deformation is present. These twin boundaries take the form of parallel sided lamellae, as shown in Figure 6, and are often called coherent twin boundaries (CT) [45]. Thus, the conclusion is drawn that the twin boundaries indicated throughout the joints of Figure 6 are a mixture of deformation/mechanical and annealing twins, both important for providing strength to the welds [46,47,48].

#### 3.2.2. Electron Backscatter Diffraction Analysis

The OM comparison of the microstructure between the identified weld regions—BM, HAZ, TMAZ, and WZ—revealed that the copper grains have undergone various degrees of refinement, with grain size decreasing in the TMAZ and WZ areas. EBSD analysis was used to assess grain morphology and recrystallisation during HFW, with results provided in Figure 7, Figure 8 and Figure 9. The increased high-angle grain boundaries (HAGBs) fraction is consistent with severe plastic deformation at elevated temperatures and indicates the operation of dynamic recrystallisation (DRX) [45,49] during HFW. Evidence of recrystallisation is further supported through the resulting GOS and KAM maps in Section 3.2.3.

The band contrast image along with the misorientation angle distributions maps of the different zones of the optimum weld C1875 are illustrated in Figure 7. Grain boundaries between 0° and 2° are not considered as LAGBs, as they may be induced due to the presence of strain and the local lattice curvature. Additionally, grain boundaries between 2° and 15° are considered substructured. The frequency of the substructured, deformed and recrystallised grains changes with the growing distance from the weld centre, indicating that the copper microstructure has undergone significant changes.

The average grain size in the BM was measured at ≈21.33 µm, and the misorientation angle distribution exhibited ≈33% of LAGBs (2–15°) and 67% of HAGBs (15–63°), respectively. LAGBs are thermally and mechanically unstable and undergo changes under the heat and strain inducted by HFW [20]. This is confirmed by the average grain size in the WZ at ≈13 µm, corresponding to a 39% decrease, and in the TMAZ, at 12.45 µm, corresponding to a 41.6% decrease. Similarly, the increased HAGBs content suggests that the recrystallised volume has also increased, reaching ≈91.7% in the WZ and ≈92.9% in the TMAZ. In the WZ, the combination of stored strain energy and the elevated temperature generated during HFW provided the necessary driving force to overcome the recrystallisation threshold, thereby promoting strain-induced DRX mechanisms. A small volume of strain energy remains in the microstructure in the form of dislocation cell structures, manifested as LAGBs (≈7.1% for TMAZ and ≈8.3 for the WZ).

#### 3.2.3. Grain Orientation Spread + Kernel Average Misorientation Analysis

Refined metallic crystals in the WZ imply the occurrence of GDRX [45] and can be identified through a grain orientation spread (GOS) map. GOS maps are a valuable primary strain analysis tool that allows one to distinguish deformed and recrystallised (i.e., strain-free) grains [49,50]. They have the capability to estimate stored strain energy during ongoing deformation in a weld [50,51,52]. Harshavardhana et al. [50] investigated the cutoff point for delineating the deformed strain-free angles for pure ETP Cu in GOS maps. In this study, it was reported that a GOS cutoff value of 1.1° was suitable for distinguishing the strain free grains from the deformed grains of pure copper even with low deformation percentage samples. Kernel average misorientation (KAM) maps provide a quantitative measure of local crystallographic misorientations, with high values indicating regions of high strain [50,53]. KAM maps are constructed by computing the average misorientation at each point with respect to its neighbours [50]. In the study by Harshavardhana et al. [50], misorientations larger than 3° were regarded as grain boundaries and were excluded in the calculations of the average misorientations. The combination of GOS and KAMP maps allowed for the complete mapping of the extent of recrystallisation.

Figure 8b,c revealed a large fraction of HAGBs (under the condition of GOS < 1.1° as per Harshavardhana et al. [50]) for weld specimen C1875, confirming the presence of strain-free recrystallised grains in the WZ and, to a similar extent, in the TMAZ. Comparatively, the GOS map for the BM in Figure 8a, shows grain regions with a higher average strain, due to the presence of deformed grains. This is due to an increase in the plastic strain inside the grains since the as-received copper has undergone cold work to increase its strength. In parallel, the KAM map in Figure 9 revealed strain concentration at the interface of grain boundaries, where the stress concentration associated with high KAM values is highlighted in green. Furthermore, the higher hardness of the as-received copper alloy is consistent with strain hardening caused by prior cold work deformation. In contrast, low KAM values are an indication that the grains are strain free and have been exposed to DRX mechanisms [45]. The KAM values presented in Figure 9b,c further support this statement, with maps being predominantly composed of blue regions and free of large volumes of green pixels, which are attributed to deformation via strain hardening/cold working.

## 4. Conclusions

The ability of hybrid friction welding in producing welds with 98.1% joint efficiency was investigated. Solid cylindrical rods of ETP Cu were rotary friction welded while a programmable brake was implemented at various rotational speed increments, avoiding solidification defects and monitoring flash development. The welds were subjected to metallographic and mechanical property investigations to assess the joint integrity. The key findings of this study are summarised as follows:

- The introduced hybrid friction welding cycle, via the application of a programmable brake, significantly improved joint efficiency, thereby recording 98.1% efficiency for weld C1875. To the best of the authors’ knowledge, no other published study has previously reported this achievement.
- Weld C1875 achieved the highest ultimate tensile strength (≈274 MPa), a 7.87% increase over the base material, and the greatest elongation (~22%) with fractography indicating a ductile fracture with visible necking. In comparison, welds (C1850, C1900) showed reduced UTS and ductility, with C1900 exhibiting brittle fracture characteristics with inactive regions at the weld interface.
- Regarding the hardness profile of weld C1875, a significant decrease to ~48–50 HV from the 80–90 HV (cold-worked as received material) was reported, followed by a slight increase from the Hall–Petch effect in the refined grains in WZ.
- EBSD analysis indicated extensive recrystallisation where grain size reduced from ~21.3 µm (BM) to ~13 µm (WZ), a 39% decrease; high angle grain boundaries increased to >91% in WZ and TMAZ, indicating strain-free grains and low angle grain boundaries decreased significantly, showing reduced stored strain energy.
- GOS and KAM maps validated the existence of DRX mechanisms: WZ exhibited predominantly strain-free grains (GOS < 1.1°), confirming full recrystallisation, whereas KAM maps showed minimal local misorientation in WZ, contrasting with high strain in BM. The presence of coherent twin boundaries varied per weld zone, with an increased percentage at TMAZ and WZ.

## Figures and Tables

**Figure 1:**
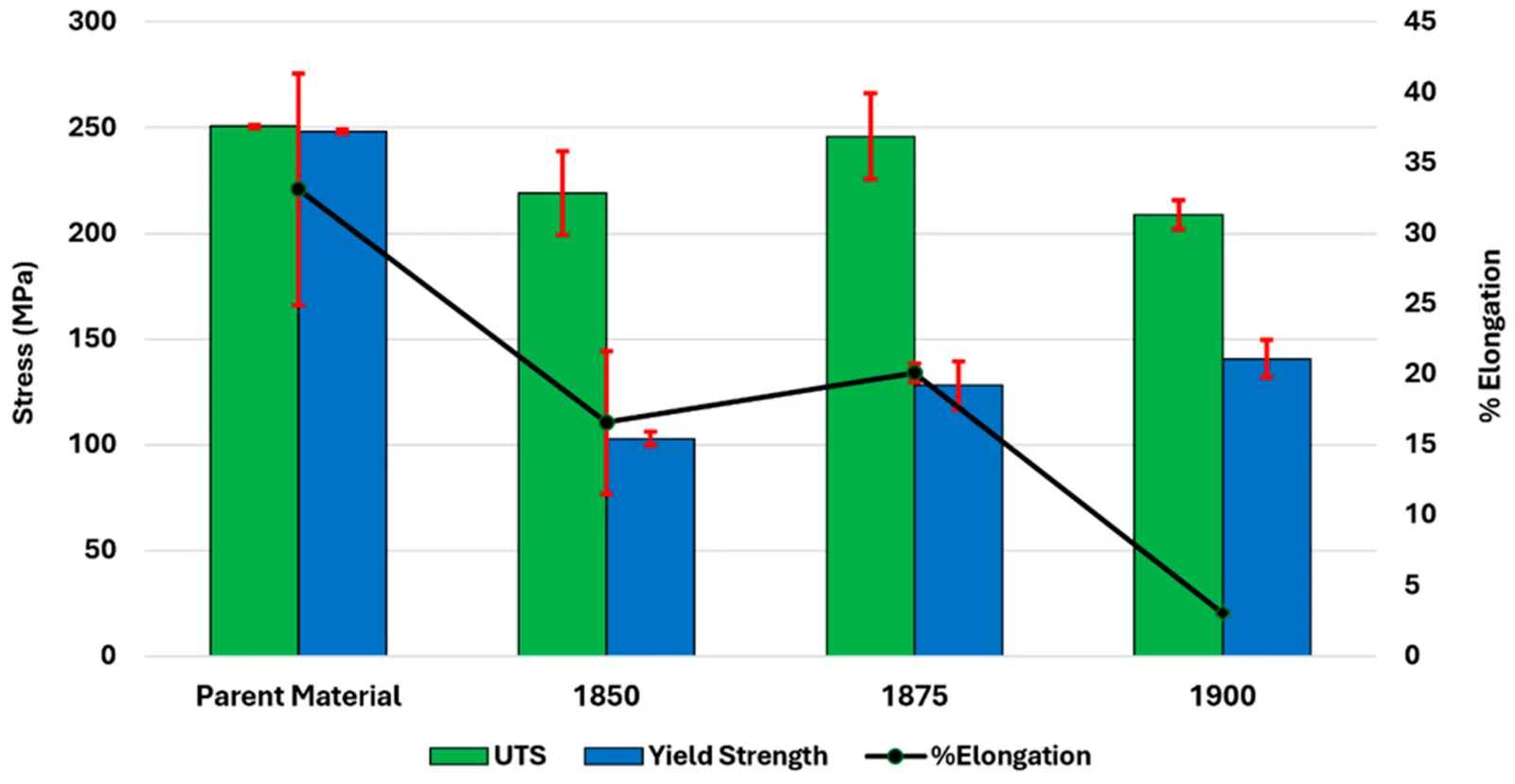
Averaged tensile behaviour—UTS, YS, and % elongation—with statistical average deviation illustrated as error bars for all welds and parent material.

**Figure 2:**
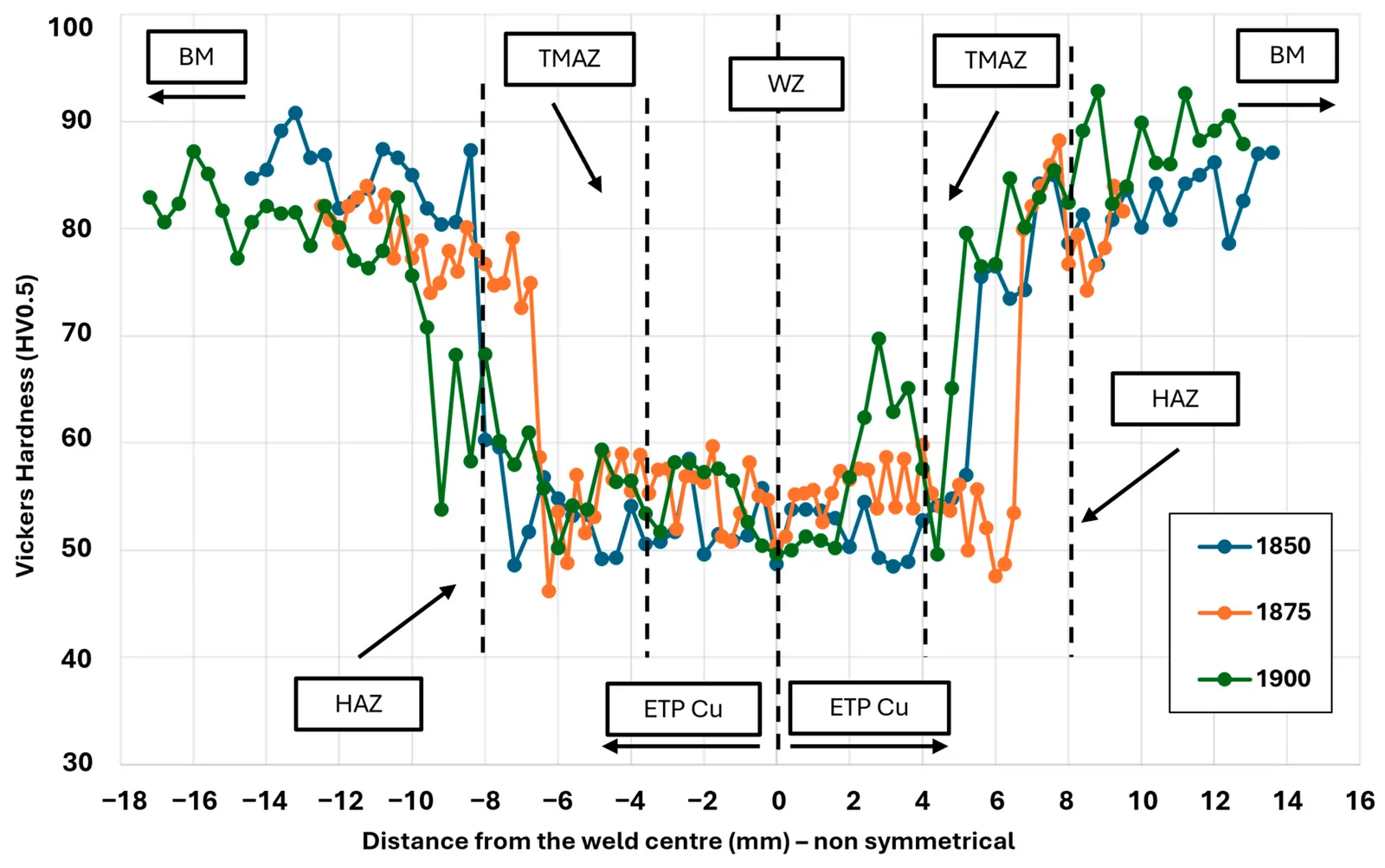
Microhardness evolution along the length of welded samples (C1900, C1875 and C1850) for all 3 stages of breaking in the centre region.

**Figure 3:**
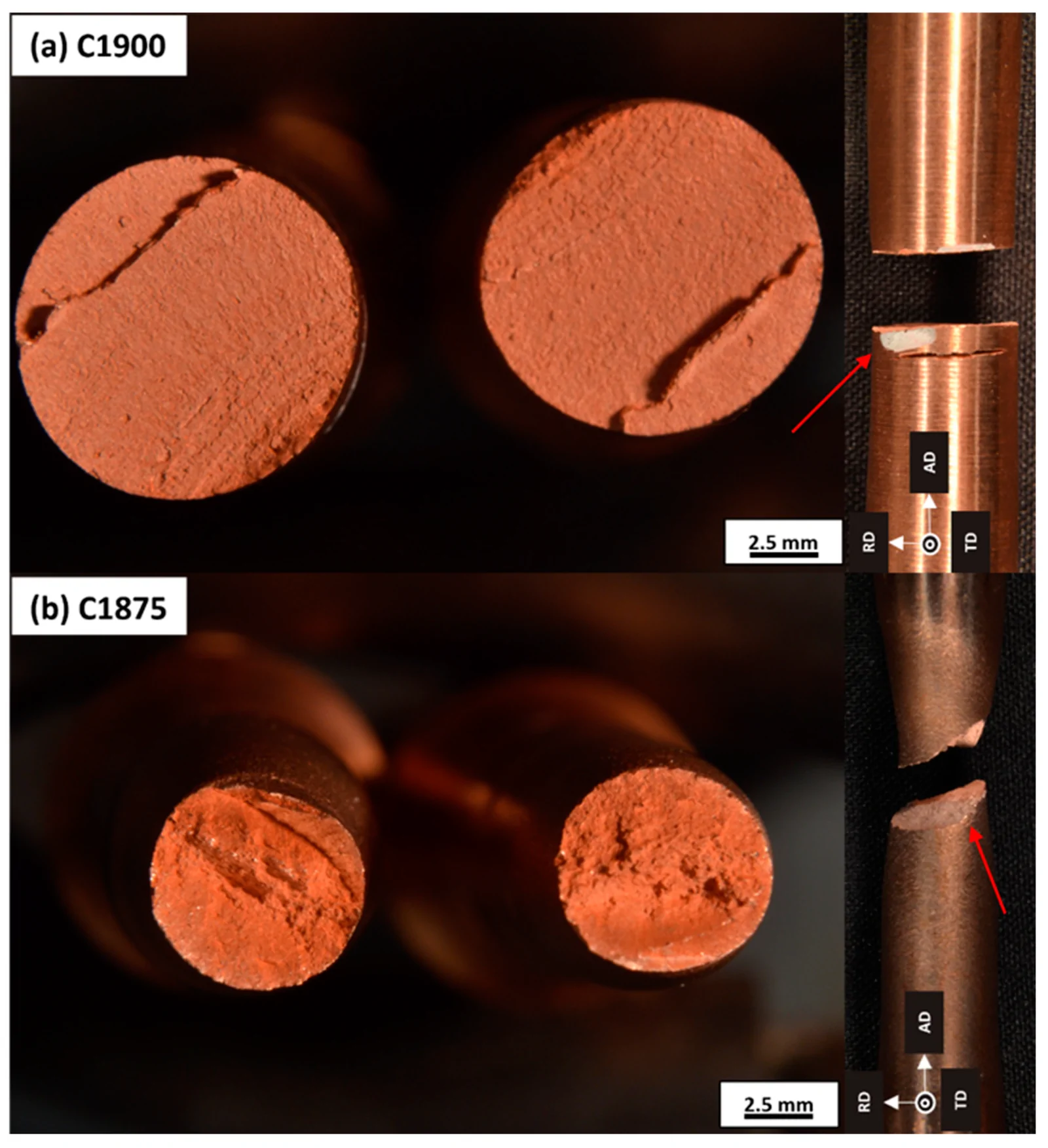
Macroscopic appearance of fractured tensile samples [(**a**) C1900, (**b**) C1875] uniaxial tension. Sample (**a**) brittle fracture and sample (**b**) ductile fracture. AD, TD and RD refer to the axial, radial and transverse directions, respectively. Red arrows indicate the estimated pre-weld copper substrate interface.

**Figure 4:**
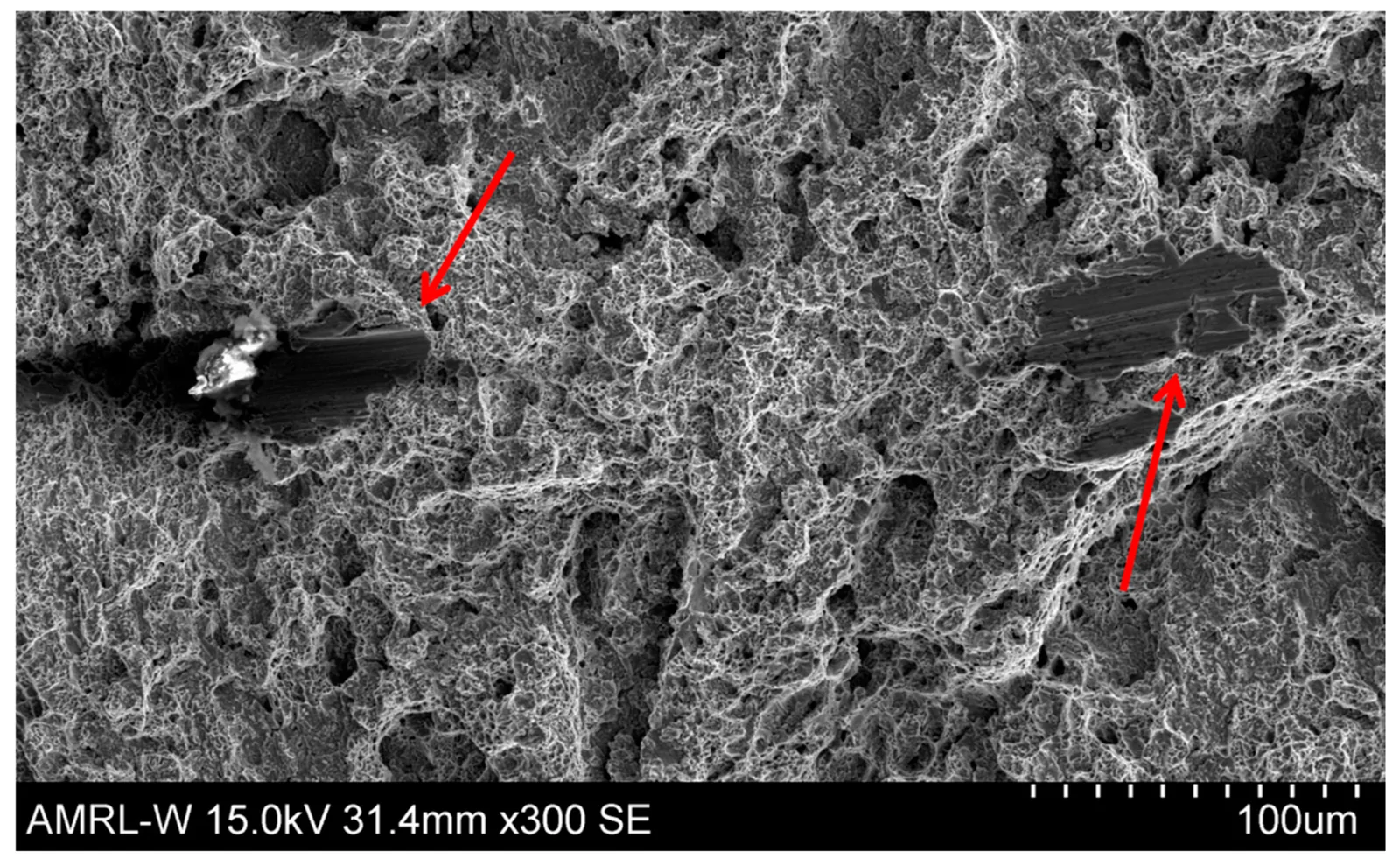
SEM image of the fractured surface for weld C1900 showing a combination of fracture mechanisms at the microscopic scale under uniform tensile loads. Red arrows indicate the non-welded flat regions.

**Figure 5:**
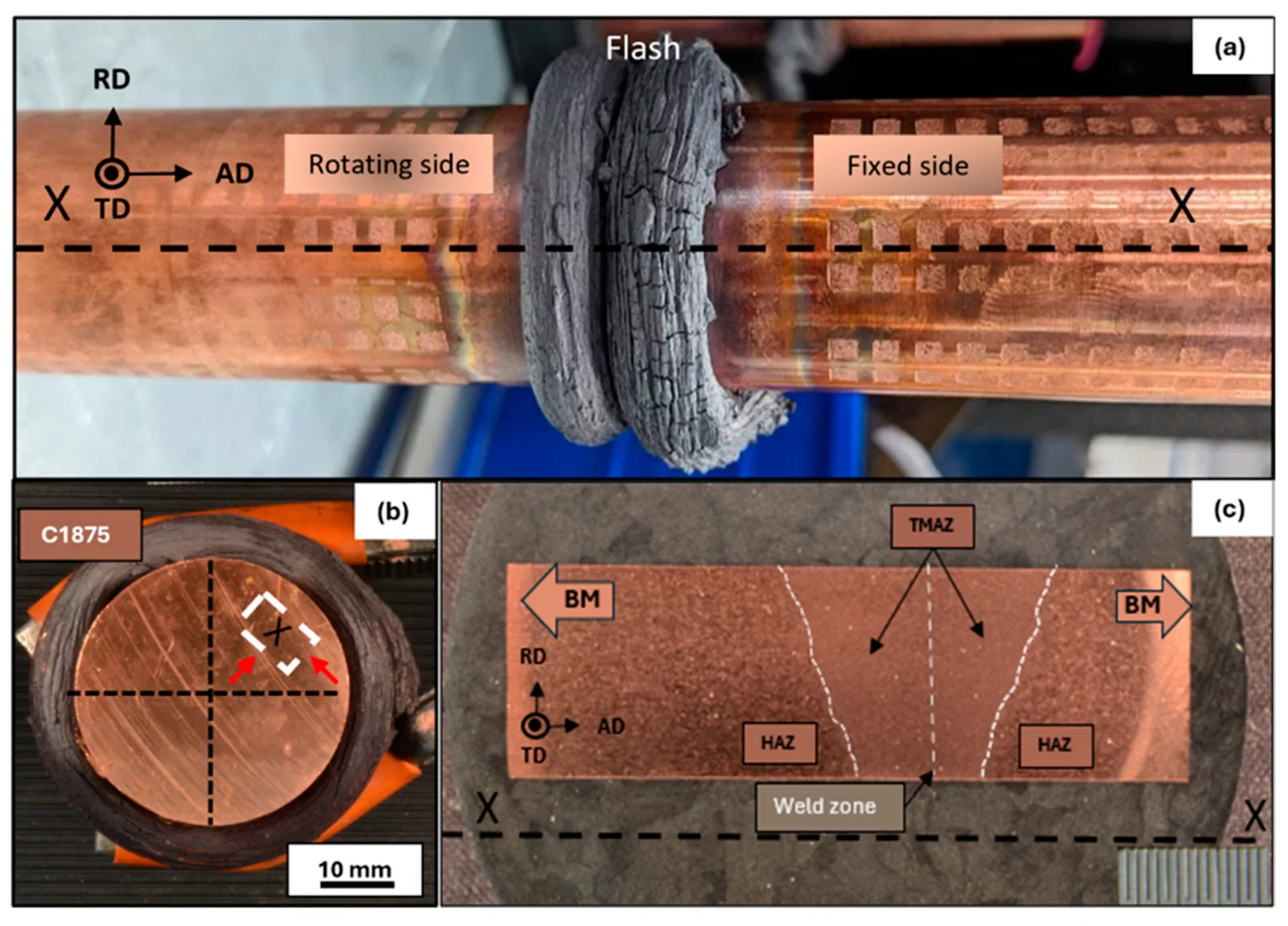
(**a**) Macrograph of the C1875 HFW joint exhibiting the rotating side, fixed side and the flash (not to scale); (**b**) cross-sectional macrograph of the weld [perpendicular of the X-X axis] top view of the weld joint; (**c**) mounted specimen used for metallographic investigation.

**Figure 6:**
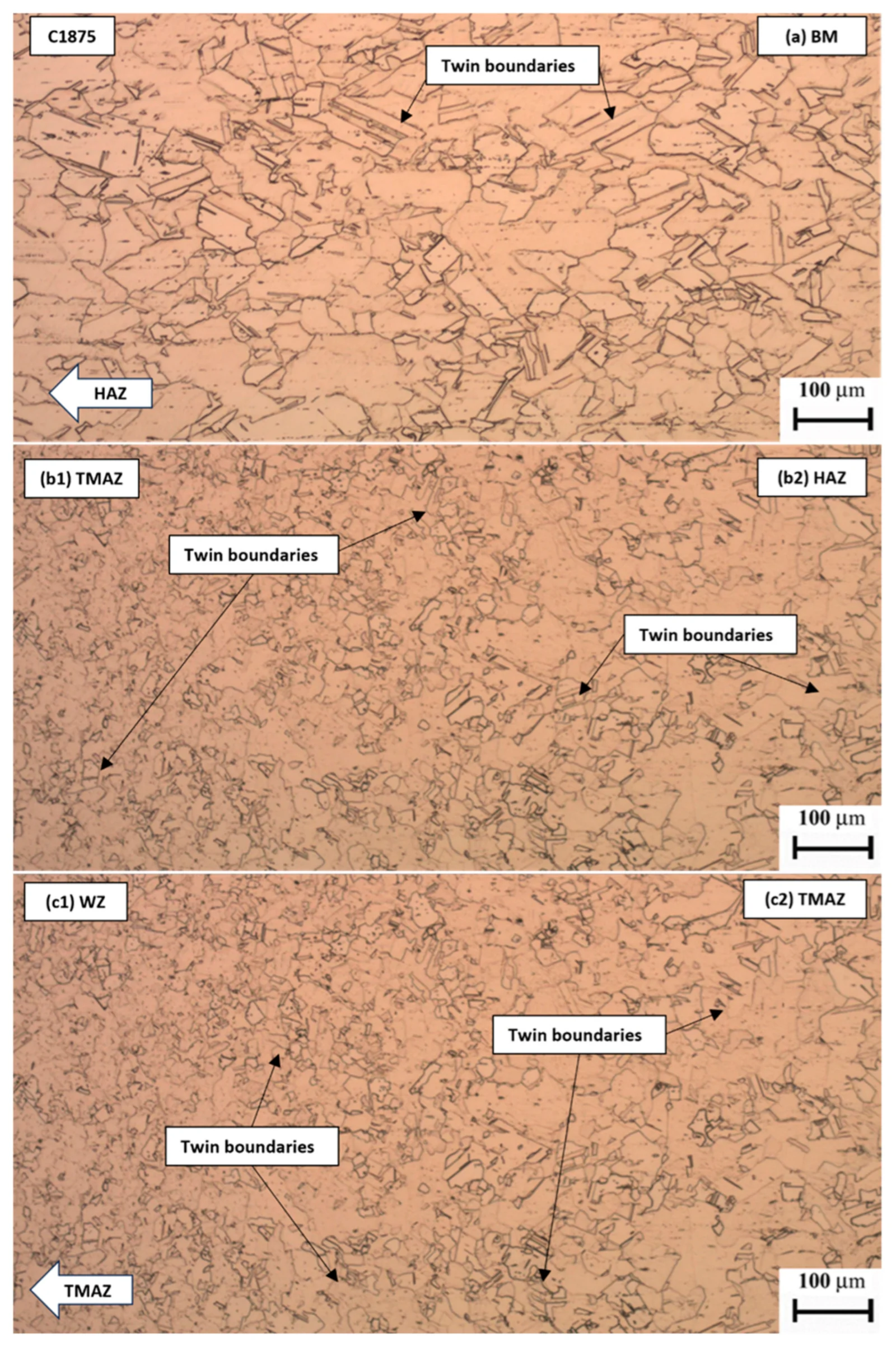
C1875 weld [×100, etched] microstructure alongside the AD where (**a**) BM, (**b1**) HAZ, (**b2**,**c2**) TMAZ, and (**c1**) BM with twin boundary annotations along the centre line of the weld zones.

**Figure 7:**
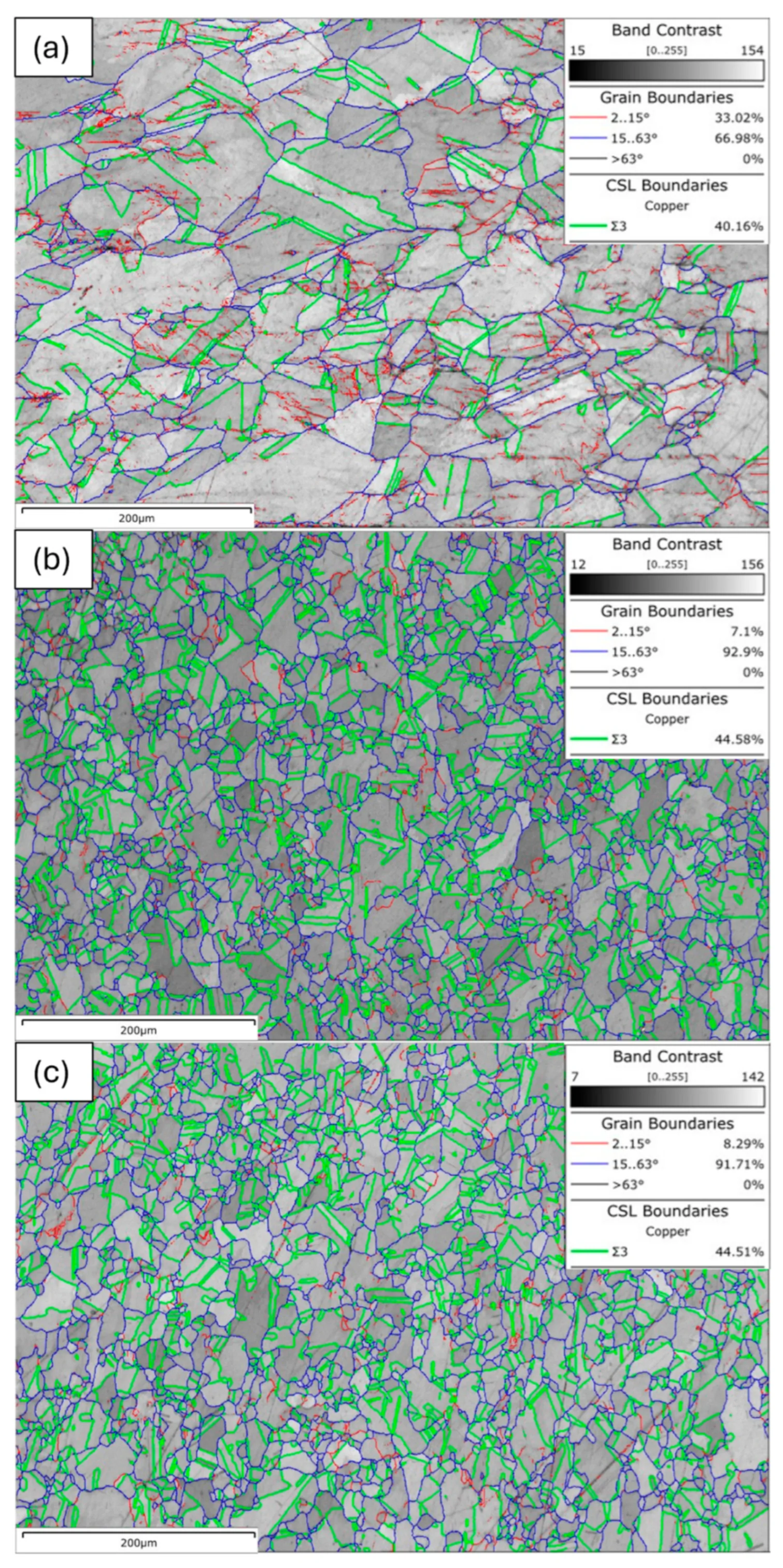
Misorientation angle distribution maps for (**a**) BM, (**b**) TMAZ and (**c**) WZ for C1875 respectively (red line: 2° < θ < 15°, blue line: 15° < θ < 63°, green line: CSL twin boundaries).

**Figure 8:**
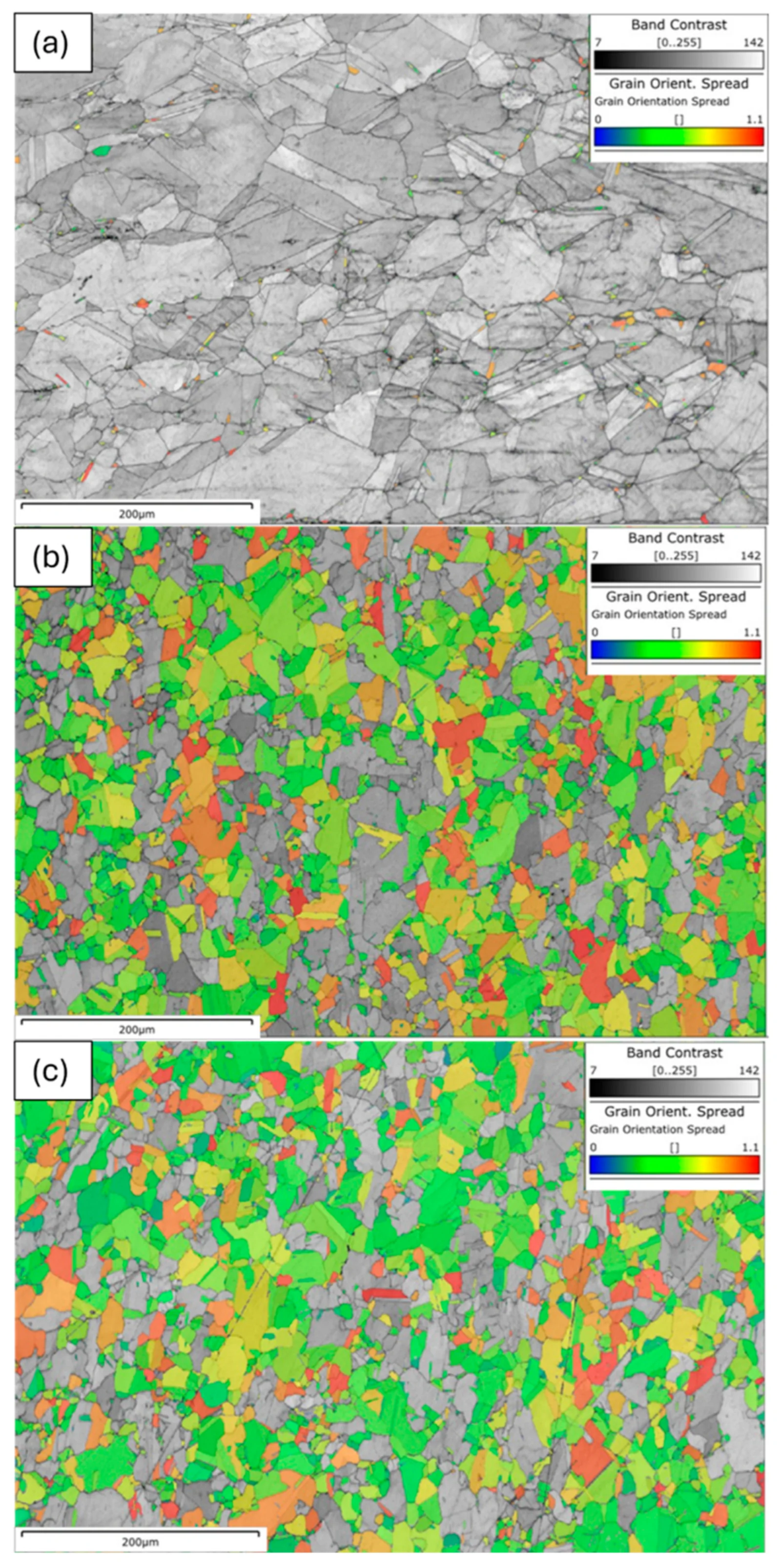
Comparison of GOS maps for (**a**) BM—deformed, (**b**) TMAZ—partially recrystallised, and (**c**) WZ—fully recrystallised samples for C1875. The misorientation spread >1.1° is marked with grey regions.

**Figure 9:**
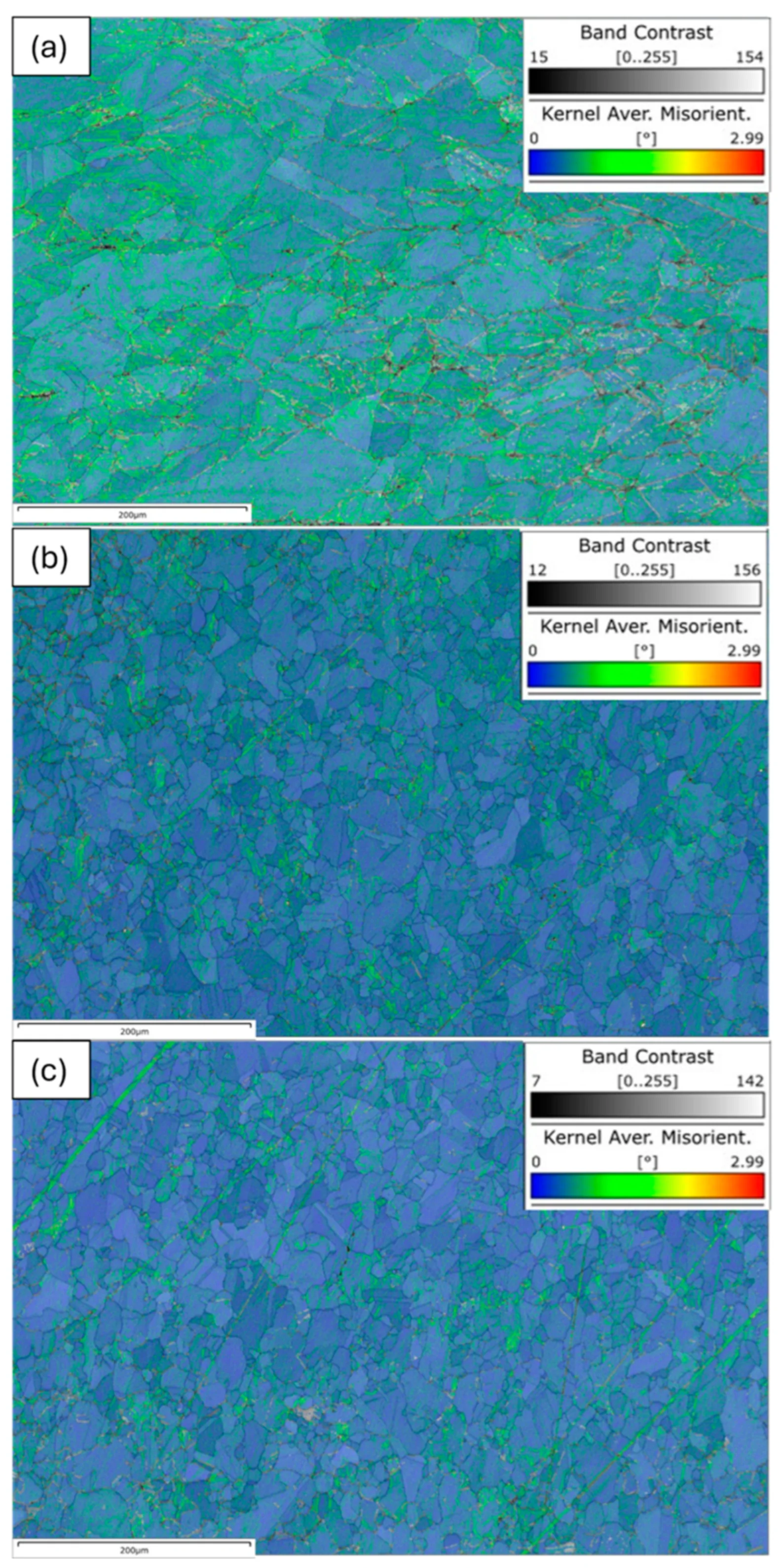
KAM distribution for weld specimen C1875: (**a**) BM—deformed, (**b**) TMAZ—partially recrystallised, and (**c**) WZ—fully recrystallised. The misorientation angle cutoff point is 3°.

**Table 1:** HFW parameters of ETP Cu solid cylindrical joints.

Weld No.	Rotational Speed (RPM)	Implemented Break (RPM)	Friction Pressure (Bar)	Forging Pressure (Bar)	Calculated Upset (mm)
1	2000	1850	5	5	4.78
2	2000	1875	5	5	4.78
3	2000	1900	5	5	4.78

## Data Availability

The original contributions presented in this study are included in thearticle. Further inquiries can be directed to the corresponding authors.
